# A new diminutive species of bohaiornithid enantiornithine (Aves: Ornithothoraces) from the Lower Cretaceous Jehol Group, northern China

**DOI:** 10.1038/s41598-024-82869-8

**Published:** 2024-12-28

**Authors:** Caizhi Shen, Alexander D. Clark, Hui Fang, Shaokun Chen, Hongxia Jiang, Qiang Ji, Jingmai K. O’Connor

**Affiliations:** 1 Hebei International Joint Research Center for Paleoanthropology, College of Earth Science, Hebei GEO University, 136 Huai’an East Road, Yuhua District, Shijiazhuang, 050031 China; 2 Negaunee Integrative Research Center, Field Museum, 1400 S. Dusable Lake Shore Drive, Chicago, IL 60605 USA; 3 Committee on Evolutionary Biology, University of Chicago, Chicago, IL 60637 USA

**Keywords:** Taxonomy, Palaeontology

## Abstract

Enantiornithes are the most successful early-diverging avian clade, their fossils revealing important information regarding the structure of Cretaceous avifaunas and the parallel refinement of flight alongside the ornithuromorph lineage that includes modern birds. The most diverse recognized family of Early Cretaceous enantiornithines is the Bohaiornithidae, known from the Jehol Biota in northeastern China. Members of this clade enhance our understanding of intraclade morphological diversity and elucidate the independent evolution of this unique lineage. Here, we report on a new specimen of bohaiornithid, *Neobohaiornis lamadongensis* gen. et sp. nov., from the Lower Cretaceous Jiufotang Formation in western Liaoning, China. The holotype specimen is considerably smaller than all other known bohaiornithids (roughly half the size of *Bohaiornis*). The presence of complete fusion in compound elements strongly suggests it represents a mature or nearly mature individual, and therefore substantially increases the known size range of this clade. This specimen further differs from known bohaiornithids in that it exhibits reduced manual unguals and an increased number of sacral vertebrae, which indicates bohaiornithids evolved increased flight capabilities in parallel to other enantiornithine lineages, such as the Longipterygidae. Traces of the plumage, which are rarely preserved in bohaiornithids, reveal the presence of remiges with rounded distal margins and short crural feathers.

**Supplementary Information:**

The online version contains supplementary material available at 10.1038/s41598-024-82869-8.

## Introduction

The most diverse Mesozoic clade of birds was the Enantiornithes^1–5^. With over one hundred named genera described to date, fossils referrable to this group of predominantly arboreal birds have been described from nearly all continents and account for half the known Mesozoic avifauna^6,7^. Although restricted to smaller body sizes in the Early Cretaceous, by the Late Cretaceous Enantiornithes achieved a significant size range and occupied a diversity of ecological niches^2,6,8^.

The greatest number of enantiornithine fossils come from deposits that record the Early Cretaceous Jehol Biota in northeastern China, accounting for slightly more than half of the known Mesozoic avian diversity (~ 130 − 120 Ma)^9^. These volcanolacustrine deposits record fossils in exceptional fidelity, preserving soft tissues and ingested remains that reveal a wealth of data concerning the biology of enantiornithines^9,10^. Several clades of enantiornithines are recognizable: the Longipterygidae, Pengornithidae, and Bohaiornithidae^11–18^. The Bohaiornithidae exhibits the greatest diversity with at least seven known genera: *Longusunguis*^17^, *Parabohaiornis*^17^, *Beiguornis*^19^, *Bohaiornis*^20^, *Sulcavis*^21^, *Shenqiornis*^22^, *Zhouornis*^23^, and possibly *Gretcheniao*^24^.

Bohaiornithids are characterized by a distinct robust tooth morphology^17^. The teeth are apically pinched, gently apicodistally curved, and mesiodistally robust near the basal portion. The rostral-most teeth are typically reduced in size compared to the remainder of the tooth row. Like most enantiornithines, bohaiornithids were likely arboreal based on their pedal morphology^6^, in which the pedal phalanges elongate distally within each digit ending in large, recurved unguals. However, compared to other taxa their claws were proportionately larger and more recurved. The largest members of the clade (*Bohaiornis*) likely approached similar sizes to extant diminutive accipitrids (~ 200 g). The largest known bohaiornithids are 167% the size of the smallest previously described specimen, an unnamed juvenile (CUGB-P1202)^1,25^.

As one of the most diverse families of enantiornithines yet described, bohaiornithids can also be considered one of the most successful Early Cretaceous avian lineages. Thus, it is reasonable to infer that this lineage of early-diverging birds independently evolved adaptations for refined flight capabilities, as observed in other diverse Jehol lineages such as the Longipterygidae^14^. Here we describe a new late immature-mature bohaiornithid enantiornithine (MHGU-0288) from the Jehol avifauna (Jiufotang Formation, ~ 119 My^26^) representing a new taxon, which adds to the considerable known bohaiornithid diversity. The new specimen preserves some new details of the plumage of bohaiornithids and a reduced alular digit, which highlights the parallel and repeated evolution of manual reduction in birds during this early stage in their evolution.

## Results

### Systematic paleontology

Aves Linnaeus, 1758; [sensu Sereno, 1999]^27^.

Ornithothoraces Chiappe, 1995.

Enantiornithes Walker, 1981.

Bohaiornithidae Wang et al., 2014.

**Fig. 1:**
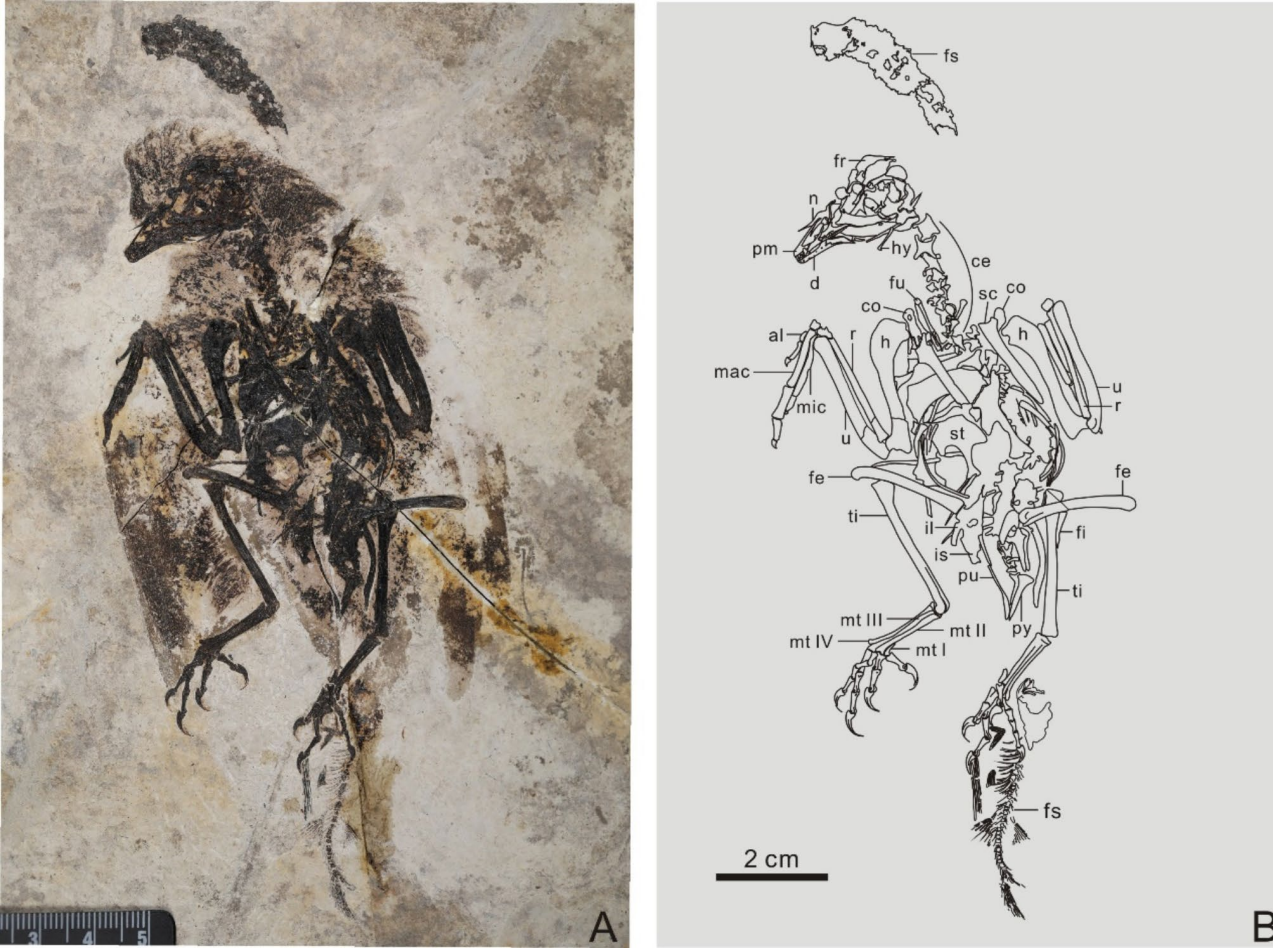
Holotype of *Neobohaiornis lamadongensis* gen.et sp. nov. (MHGU-0288). (**A**) Photograph; (**B**) line drawing. *al* alular metacarpal, *ce* cervical vertebrae, *co* coracoid, *d* dentary, *fr* frontal, *fe* femur, *fi* fibula, *fs* fish, *fu* furcula, *h* humerus, *hy* hyoid, *il* illium, *is* ischium, *mac* major metacarpal, *mic* minor metacarpal, *mt I-IV* metatarsal I-IV, *n* nasal, *pm* premaxilla, *pu* pubis, *py* pygostyle, *r* radiale, *sc* scapula, *st* sternum, *ti* tibiotarsus, *u* ulna.

*Neobohaiornis lamadongensis* gen. et sp. nov. (Fig. 1)

## Etymology

The generic name refers to the derived morphology (e.g., reduced alular digit, increased number of sacral vertebrae) of this taxon relative to other bohaiornithids. The specific name refers to the town of Lamadong, near where the fossil was found.

## Holotype

MHGU-0288 (housed at the Museum of Hebei GEO University), a nearly complete and articulated skeleton preserved in dorsal aspect with traces of feathers surrounding the skeleton (Fig. 1; Table 1).

**Table 1:** Measurements (in mm) of *Neobohaiornis lamadongensis* gen.et sp. nov. (MHGU-0288).

Element	Left	Right
Skull	26.7	
Sternum	17.5*	
Furcular ramus	10.0	
Hypocleidium	–	–
Coracoid length	6.4*	6.8*
Coracoid distal width	14.3*	13.7*
Scapular	16.3*	19.0*
Humerus	25.4	25.3
Radius	21.5	22.1
Ulna	25.7	26
Metacarpal I	2.3	
Manual digit I-1	4.2	
Manual digit I-2	1.5	
Metacarpal II	11.6	12.4
Manual digit II-1	6.4	
Manual digit II-2	3.8	
Manual digit III-3	1.7	
Metacarpal III	13.1	13.4
Manual digit III-1	3.1	3.3
Manual digit III-2	0.4*	
Pygostyle	9.3	
Ilium	7.9*	10.2*
Pubis	19.1	19.6
Ischium	7.6*	8.4*
Synsacrum	13.8	
Femur	22.2	22.1
Tibiotarsus	27.4	27.2
Fibula	3.8*	6.7*
Metatarsal I	2.8	2.9
Pedal digit I-1	4.3	4.5
Pedal digit I-2	4.5	4.0
Metatarsal II	13.2	11.6*
Pedal digit II-1	3.3	3.5
Pedal digit II-2	4.2	4.3
Pedal digit II-3	4.4	4.3
Metatarsal III	14.4	14.4
Pedal digit III-1	4.8	4.9
Pedal digit III-2	4.1	4.0
Pedal digit III-3	4.1	4.1
Pedal digit III-4	4.5	4.4
Metatarsal IV	14.0	13.6
Pedal digit IV-1	2.5	2.2*
Pedal digit IV-2	2.5	2.3
Pedal digit IV-3	2.4	2.3
Pedal digit IV-4	3.4	3.1
Pedal digit IV-5	3.0	3.4

* indicates preserved length.

## Locality and horizon

Near Lamadong, Jianchang, Liaoning Province, northeastern China; Lower Cretaceous, Jiufotang Formation (~ 119 My)^26^.

## Diagnosis

Referrable to Enantiornithes based on the presence of the following diagnostic features: a furcula with a ventral margin wider than dorsal margin; a proportionately elongate hypocleidium of the furcula; minor metacarpal projecting distally farther than major metacarpal; metatarsal IV mediolaterally thinner than both metatarsals II and III with the trochlea reduced to a single condyle; and a J-shaped metatarsal I. Referred to the enantiornithine clade Bohaiornithidae based on the presence of the following diagnostic features: premaxillary and rostral-most dentary teeth being smaller in size than distal teeth; basally robust teeth with tapered and slightly recurved apices; caudolateral projection of the lateral trabecula of the sternum; distal inflation of the lateral sternal trabecula with greater medial expansion; minor metacarpal proportionately longer than most other enantiornithines; pygostyle tapered caudally (distinct distal constriction absent); and robust proximal phalanges of pedal digits I and II.

*Neobohaiornis lamadongensis* is an icterid-sized (~ 55 g) enantiornithine with the following unique combination of features: ceratobranchial bones rostrally expanded; furcular rami straight, defining a 50˚ angle; robust, medially-curved intermediate lateral trabeculae of the sternum; alular digit ending well before the distal margin of the major metacarpal; penultimate phalanx of the major digit proportionately shorter and robust; alular and major digit unguals reduced; forked cranial processes on the pygostyle strongly developed.

### Description

#### Skull

**Fig. 2:**
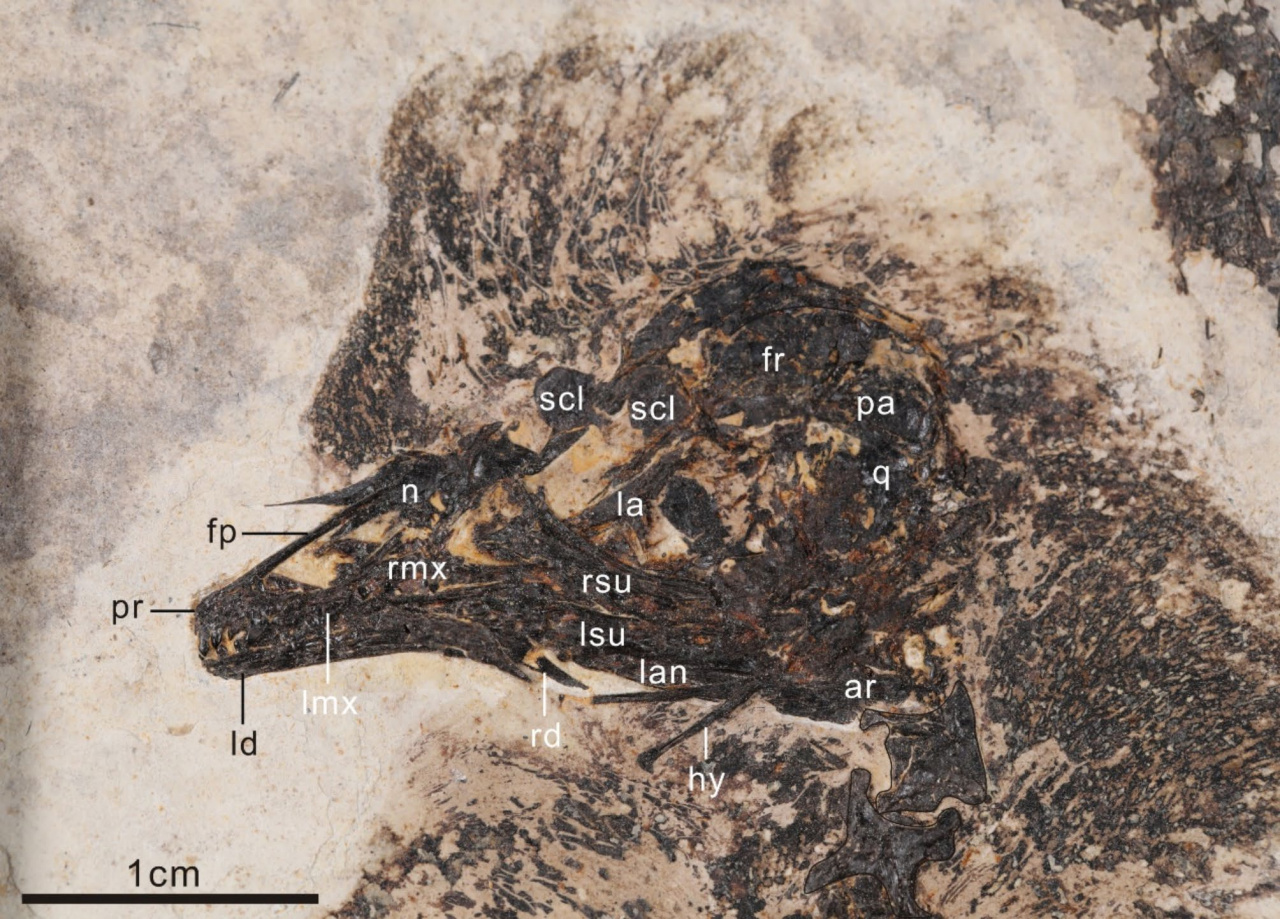
Skull of *Neobohaiornis lamadongensis* gen.et sp. nov. (MHGU-0288). *ar* articular, *fp* frontal process, *fr* frontal, *hy* hyoid, *la* lacrimal, *lan* left surangular, *ld* left dentary, *lmx* left maxilla, *lsu* left angular, *n* nasal, *pa* parietal, *pr* premaxilla, *q* quadrate, *rd* right dentary, *rmx* right maxilla, *rsu* right surangular, *scl* scleral ossicles.

The skull of *Neobohaiornis* is exposed in left lateral view (Fig. 2). The rostrum appears robust, similar to other bohaiornithids^1,13^. The angle between the frontal process and maxillary process of the premaxilla is approximately 27°, as in *Parabohaiornis* (26°)^17^, *Sulcavis* (~ 30°)^21^, *Shenqiornis* (27°)^22^, in contrast with more delicate in *Rapaxavis* (< 15°)^16^.

The maxillary process of the premaxilla is slender and straight caudally, and forms a dorsally overlapping articulation with the premaxillary ramus of the maxilla, as in *Parabohaiornis*^17^ and *Longusunguis* (IVPP V18693)^28^. The number of premaxillary teeth is uncertain, but at least three are present in situ. The premaxillary teeth are apically pinched, and basally expanded along both the distal and mesial margins as in other bohaiornithids^1,2,6^.

The dorsal process of the maxilla is concave dorsoventrally in lateral view, which participates in the ventral and caudal margin of the external naris. This process is slender and is caudodorsally oriented. At least four teeth are preserved in the left maxilla. The right and left nasals are exposed in dorsal view. The premaxillary process of the nasal is long and slender, and gradually increases in mediolateral width towards the internasal suture. These two bones are vaulted, with a ridge present on the dorsal surface. The lateral margin of this bone is concave and forms the caudal margin of the external naris. The ascending process of the maxilla is rostrocaudally 55% wider than the nasal process of the premaxilla.

The left lacrimal is well preserved. The bone is triradiate, with subequal rostral and caudal rami in length, in contrast to *Parabohaiornis*^17^ and *Zhouornis*^23^. The dorsal margin is straight at the midpoint as in *Pterygornis*^29^, different from the concave margin of *Longusunguis*^17^, *Parabohaiornis*^17^, and *Musivavis*^30^. The jugal ramus of the lacrimal is oriented ventrodorsally. A recess can be seen along the dorsal corner of the jugal ramus. The frontal is dorsally convex and forms the dorsal margin of the orbit. Two square-shaped sclerotic ossicles are preserved near the caudal portion of the orbit. A bone positioned ventral to the parietal is interpreted as the quadrate (Fig. 2). The orbital process is unclear due to overlap by other bones. The mandibular process appears bicondylar and has a concave margin, as in other enantiornithines. The oval-shaped parietals are unfused to each other.

The dentaries are unfused rostrally. The rostral portion of the left dentary exhibits approximately five in-situ teeth. The dentary teeth, while similar to those in the premaxilla are slightly less apically pinched and the crowns exhibit greater mesiodistal expansion. The dorsal and ventral margins of the dentary are straight for most of their length similar to other bohaiornithids^1,13,17^. Caudally, the ventral margin is concave and exhibits a caudoventrally sloping surangular articulation. The surangular is robust, with a caudal portion that is dorsoventrally deeper than the rostral portion (rostral portion ~ 41% that of caudal). Caudally, the lateral surface of the left surangular appears pierced by an oval foramen. The angular is ventrally convex . The retroarticular process of the articular is long with a rounded caudal margin. A pair of rod-like hyoid bones are preserved which we identify as ceratobranchials. These bones are long and straight, with a triangular shape articulation on the rostrally end that is not observed in other enantiornithines^13,29,31,32^.

## Axial skeleton

**Fig. 3:**
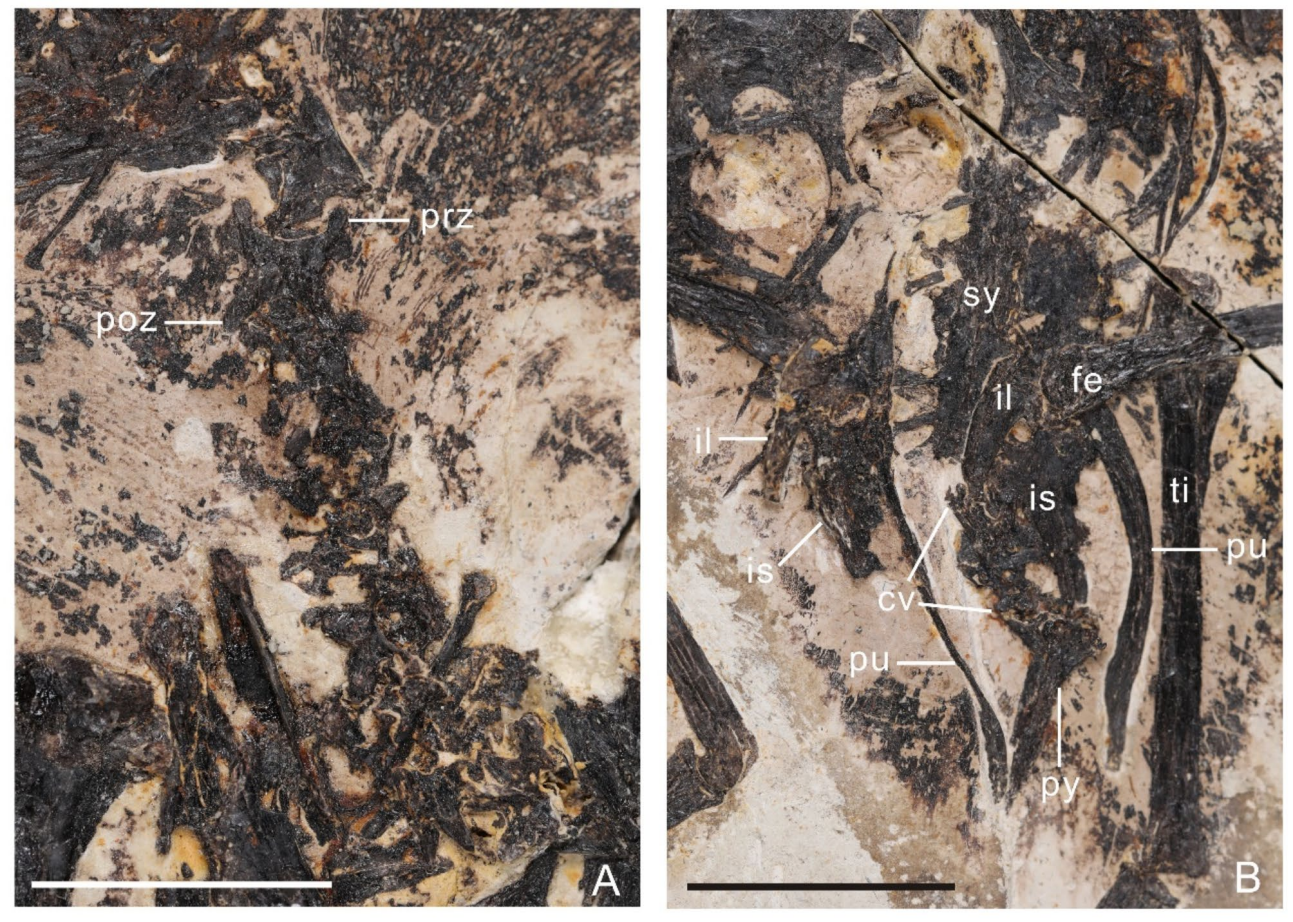
The axial skeleton of the holotype of *Neobohaiornis lamadongensis* gen.et sp. nov. (MHGU-0288). (**A**) cervical vertebrae; (**B**) caudal vertebrae. *cv* caudal vertebrae, *fe* femur, *il* ilium, *is* ischium, *poz* postzygapophysis, *prz* prezygapophysis, *pu* pubis, *py* pygostyle, *sy* synsacrum, *ti* tibiotarsus. Scale bar equals 1 cm.

At least seven cervical vertebrae are preserved in articulation (Fig. 3A). The centra of the cranial cervicals are mediolaterally wider than they are craniocaudally long, as in other bohaiornithids (e.g., *Bohaiornis*,* Longusunguis*). The prezygopophyses are craniolaterally oriented. The postzygopophyses distally taper, project caudolaterally, and bear prominent epipophyses that do not project caudally further than the postzygapophyses. The thoracic vertebrae are obscured by poor preservation. The sacral vertebrae are fused forming a true synsacrum composed of least seven vertebrae based on the observable transverse processes but up to nine based on the preserved length and accounting for missing processes, compared to six-seven in other bohaiornithids^17,19,20^(Fig. 3B). Three free caudal vertebrae are preserved (Fig. 3B). The caudal-most caudal vertebrae are fused into a pygostyle with well-developed paired craniodorsal processes. The dorsal and ventral margins are straight and the body of the pygostyle tapers caudally without the distal constriction common among bohaiornithid enantiornithines^17,20,21,23^.

### Pectoral girdle

**Fig. 4:**
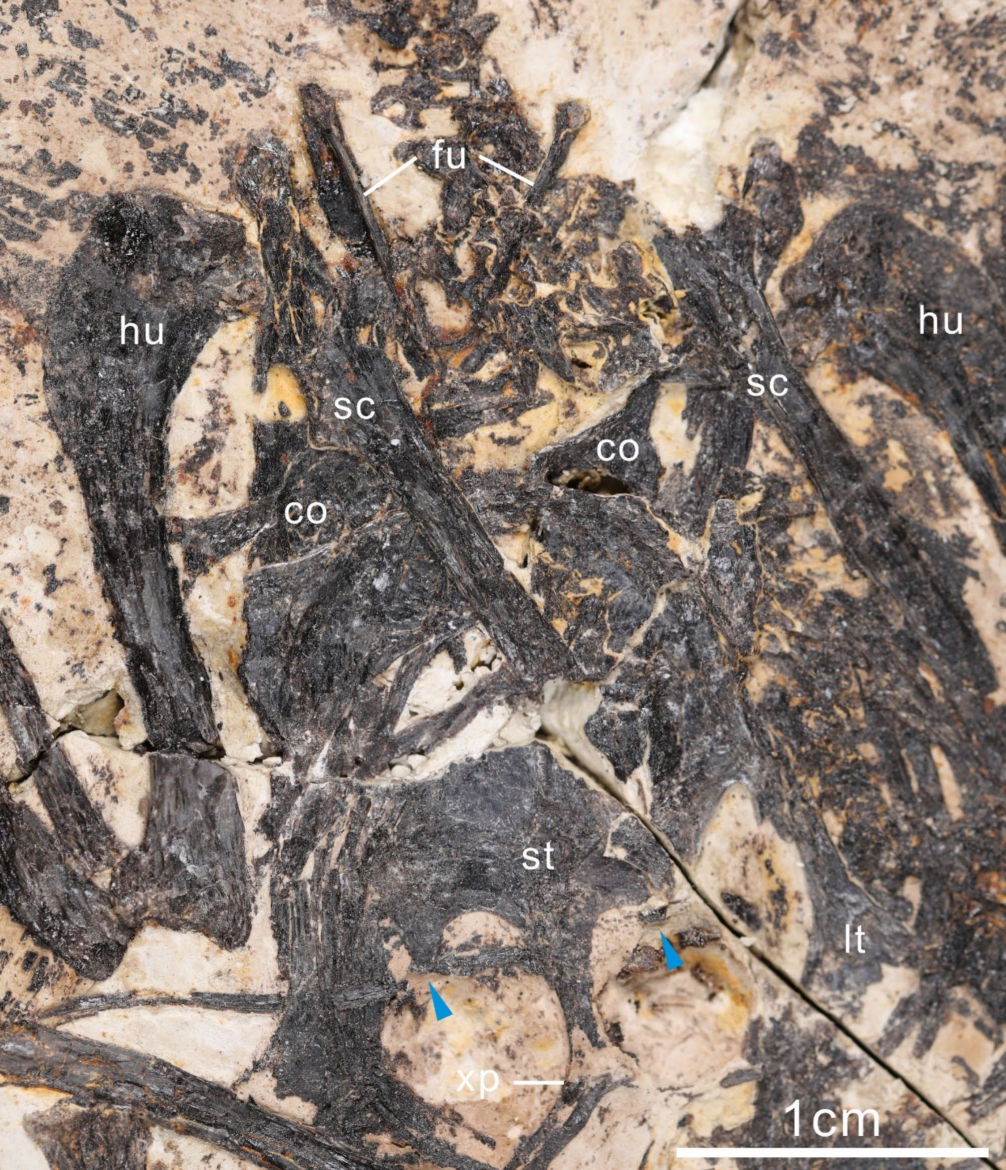
Pectoral girdle of the holotype of *Neobohaiornis lamadongensis* gen.et sp. nov. (MHGU-0288). *co* coracoid, *fu* furcula, *hu* humerus, *lt* lateral trabecular, *sc* scapula, *st* sternum, *xp* xiphoid process. The blue arrows indicate intermediate trabeculae.

The sternum is convex along the cranial margin, although less arched compared to some enantiornithines (i.e., *Rapaxavis*^16^, *Bohaiornis*^20^, *Longusunguis* IVPP V18693^28^, *Cathayornis*^33^) (Fig. 4). Craniolateral processes are present as in *Shanweiniao*^15^ and *Zhouornis*^23^. These processes are weakly developed, not projecting as far cranially as the midline of the cranial margin. The lateral trabeculae bear fan-shaped distal expansions, and are caudolaterally oriented, as in other bohaiornithids^1,13^. The intermediate trabeculae are short, robust, and curve medially. The xiphoid process terminates slightly distal to the lateral trabeculae and is weakly expanded distally with a flat caudal margin.

The scapular blades are straight and slightly caudally expanded, tapering into a blunted tip. The acromion process projects cranially, parallel to the scapular shaft. It is slightly shorter than the humeral articular facet and untapered. The strut-like coracoids have elongated necks distally expanded into a fan-shaped pterygoma, as in other enantiornithines (base of the pterygoma to just proximal to the acrocoracoidal expansion; MHGU-0288, 17%; *Parabohaiornis*, 18%^17^; *Bohaiornis*, 19%^20^; *Longipteryx*, 15%^34^; *Imparavis*, 21%^35^). The medial margins of the coracoids are concave; the lateral margins appear straight. The furcula is Y-shaped, with an interclavicular angle of ~ 50 degrees, as in *Shenqiornis* (~ 50°)^22^, but more acute than those of *Bohaiornis* (~ 65°)^1^, *Parabohaiornis* (~ 60°)^17^, *Beiguornis* (~ 62°)^19^, *Sulcavis* (~ 60°)^21^, *Longipteryx* (~ 70°)^15^. The distal portion of the right furcular rami exhibits globe-shaped epicleidial expansion also observed in other bohaiornithids (i.e., *Bohaiornis*, *Parabohaiornis*). The furcular rami are dorsolaterally excavated so that their cross-section is L-shaped and the ventral margin is much wider than the narrow dorsal margin, an autapomorphy of enantiornithines.

### Forelimb

**Fig. 5:**
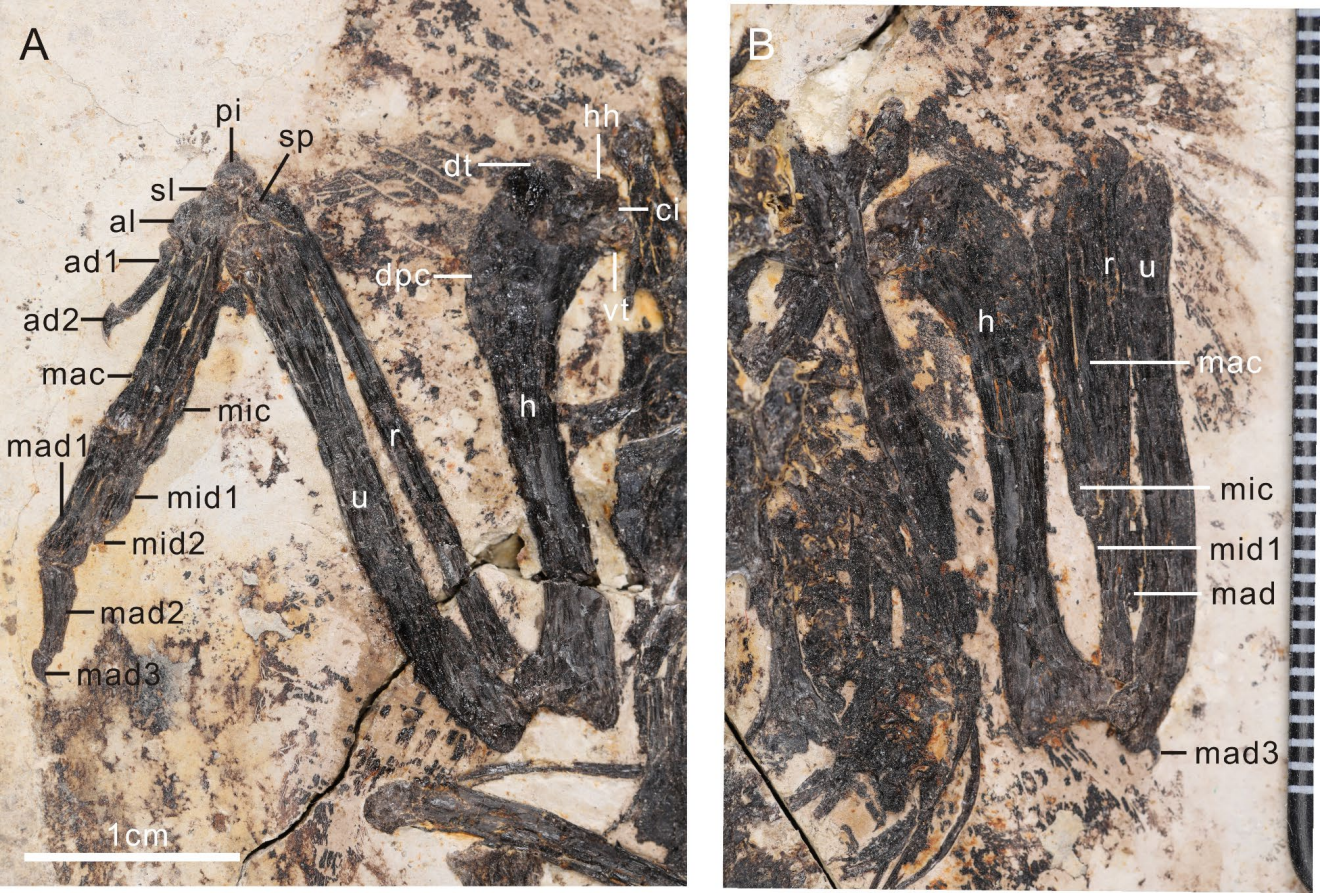
Forelimb of the holotype of *Neobohaiornis lamadongensis* gen.et sp. nov. (MHGU-0288). (**A**) left forelimb; (**B**) right forelimb. *al* alular metacarpal, *ad1-2* alular digit 1–2, *ci* capital incision, *dpc* deltopectoral, *dt* dorsal tubercle, *h* humerus, *hh* humeral head, *mac* major metacarpal, *mad1-3* major digit 1–3, *mic* minor metacarpal, *mid1-2* minor digit 1–2, *pi* pisiform, *r* radiale, *sl* semilunate carpal, *sp* scapholunare, *u* ulna, *vt* ventral tubercle.

The humerus shaft is relatively straight with both the proximal and distal margins oriented along the same plane (Fig. 5). The humerus-ulna length ratio is 98%, in contrast to 88% in *Eoalulavis*^36^. The deltopectoral crest is narrow and extends 28% of the humerus similar to other bohaiornithids^1,13^. In contrast to *Bohaiornis*, the deltopectoral crest slopes gently into the shaft distally^20^. The bicipital crest is well developed, cranially projecting, and globe shaped, similar to bohaiornithids^17^, *Gretcheniao*^24^ and *Imparavis*^35^. The humeral head is separated from the ventral tubercle by a capital incision as in other enantiornithines^6^. There is a wide and shallow groove between the dorsal tubercle and deltopectoral crest (Fig. 5A). The distal humerus is expanded relative to the shaft. The dorsal condyle is larger than the ventral condyle. The ventral tubercle is not well developed as in *Musivavis*^30^ and BMNHC-Ph1204^13^. The flexor process is weakly developed such that the distal margin of the humerus is angled relative to the long axis of the humeral shaft. The ulna is subequal to the humerus in length and more robust than the radius. The proximal portion is bowed and straight in the distal. The olecranon is weakly developed. The radius is straight, about 89% the length of the ulna, in contrast to the nearly equal length in other bohaiornithids, *Bohaiornis*^20^, *Sulcavis*^21^, *Shenqiornis*^22^ and *Musivavis*^30^. At the midpoint, the mediolateral width of the radius measures 58% that of the ulna. The scapholunare is heart-shaped. The pisiform is preserved in articulation on the top of the radius, with a saddle-shaped articulation face. The semilunate carpal is fused to the major and minor metacarpals.

The alular metacarpal is sub-rectangular shaped and about 20% the length of the major metacarpal. The alular metacarpal is unfused to the major metacarpal. The major metacarpal is wider and thicker than other metacarpals (180% and 117% deeper than the alular and minor metacarpals respectively). It is straight for most of its length except where it becomes slightly bowed distally due to the presence of a weak dorsal expansion at the distal end. The minor metacarpal is curved along its proximal half, forming a small intermetacarpal space. The minor metacarpal, like most enantiornithines^1,17,19,20^, extends distally past the termination of the major metacarpal. As in other bohaiornithids^13,17,24^, this is more developed in *Neobohaiornis* than most other enantiornithines.

The manual phalangeal formula is 2-3-2, as in most other enantiornithines^6^. The proximal end of the first alular digit is expanded and the shaft bows laterally. Phalanx I of the alular digit also measures only 36% the proximodistal length of the major metacarpal, shorter than most other enantiornithines (*Parabohaornis* 49%^17^, *Bohaiornis* 45%^20^, *Sulcavis* 62%^21^, *Shenqiornis* 46%^22^, *Longusunguis* 46%^28^, *Longipteryx* 53%^34^, *Imparavis* 51%^35^) with the exception of *Rapaxavis* (37%), in which the alular digit is reduced and lacking an alular ungual phalanx^14,16^. The claw of the alular digit is equal to the major digit claw. The major digit is very robust; the first phalanx is the longest phalanx in the hand and more robust (craniocaudally wider) than the major metacarpal. The second major digit phalanx is shorter and tapers distally. The length of major digit phalanx II compared to phalanx I is significantly reduced (59%) compared to BMNHC-Ph1204 (79%)^13^, *Parabohaiornis* (73%)^17^, *Beiguoornis* (81%)^19^, *Sulcavis* (73%)^21^, *Longipteryx* (92%)^34^ and *Imparavis* (74%)^35^. Phalanx I of the minor digit is subequal in craniocaudal depth to the minor metacarpal except where it tapers just before its articulation with the second minor digit phalanx.

### Pelvic girdle

Both ilia are preserved, but the preacetabular wings are lost (Fig. 3B). The postacetabular wing is narrow and bluntly tapers caudally. The dorsal margin of the postacetabular is convex. The right and left pubes viewed in dorsal and lateral aspects respectively, are concave dorsally and medially (convex ventrally and laterally). The pubes formed an unfused contact along the distal quarter of their lengths. The ischia are not well preserved, but the right element indicates the presence of a dorsal process that contacted the ilium, as in other enantiornithines, and a distally tapered body.

### Hindlimb

**Fig. 6:**
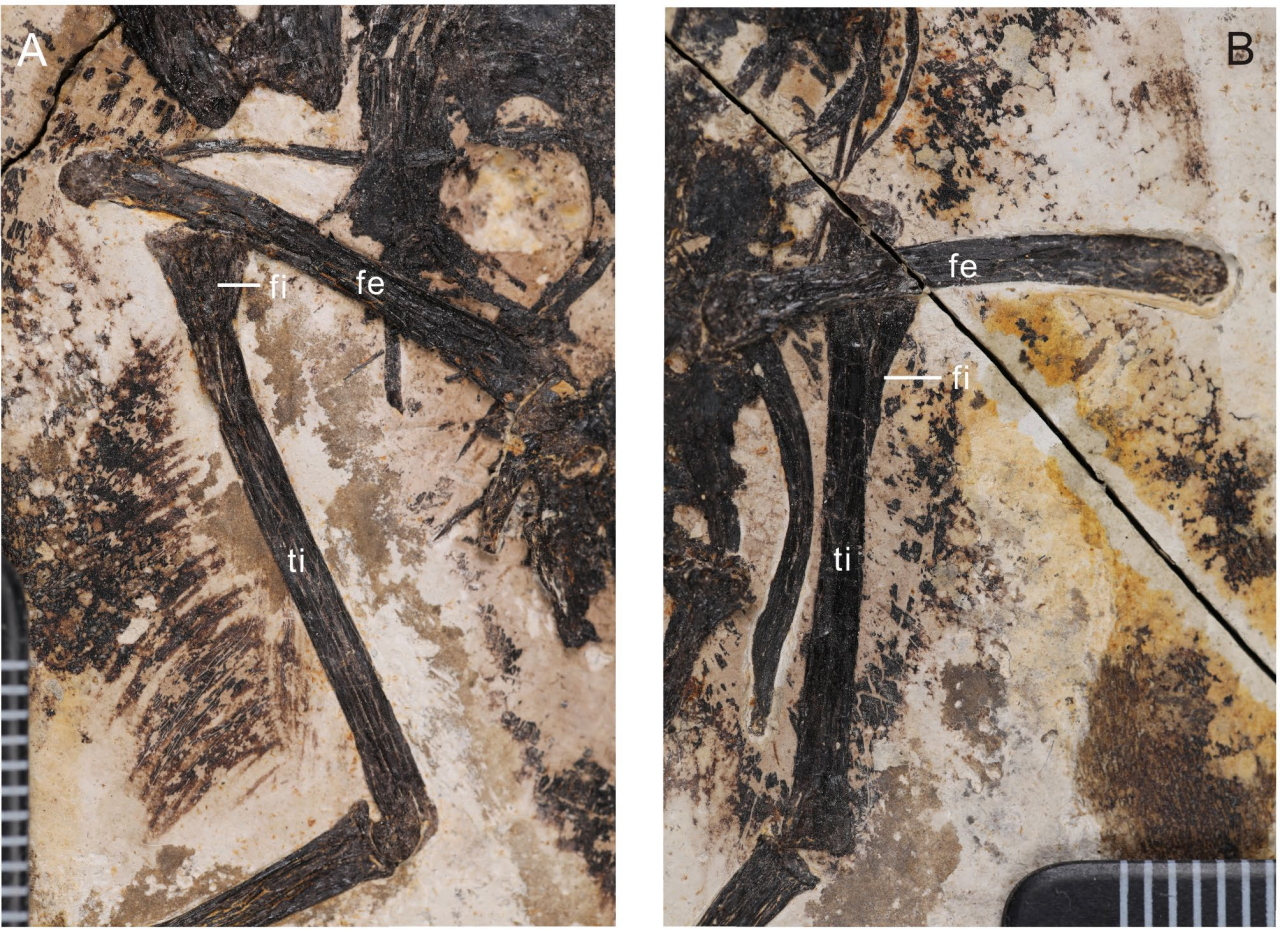
Hindlimb of the holotype of *Neobohaiornis lamadongensis* gen.et sp. nov. (MHGU-0288). (**A**) left hindlimb; (**B**) right hindlimb. *fe* femur, *fi* fibula, *ti* tibiotarsus.

**Fig. 7:**
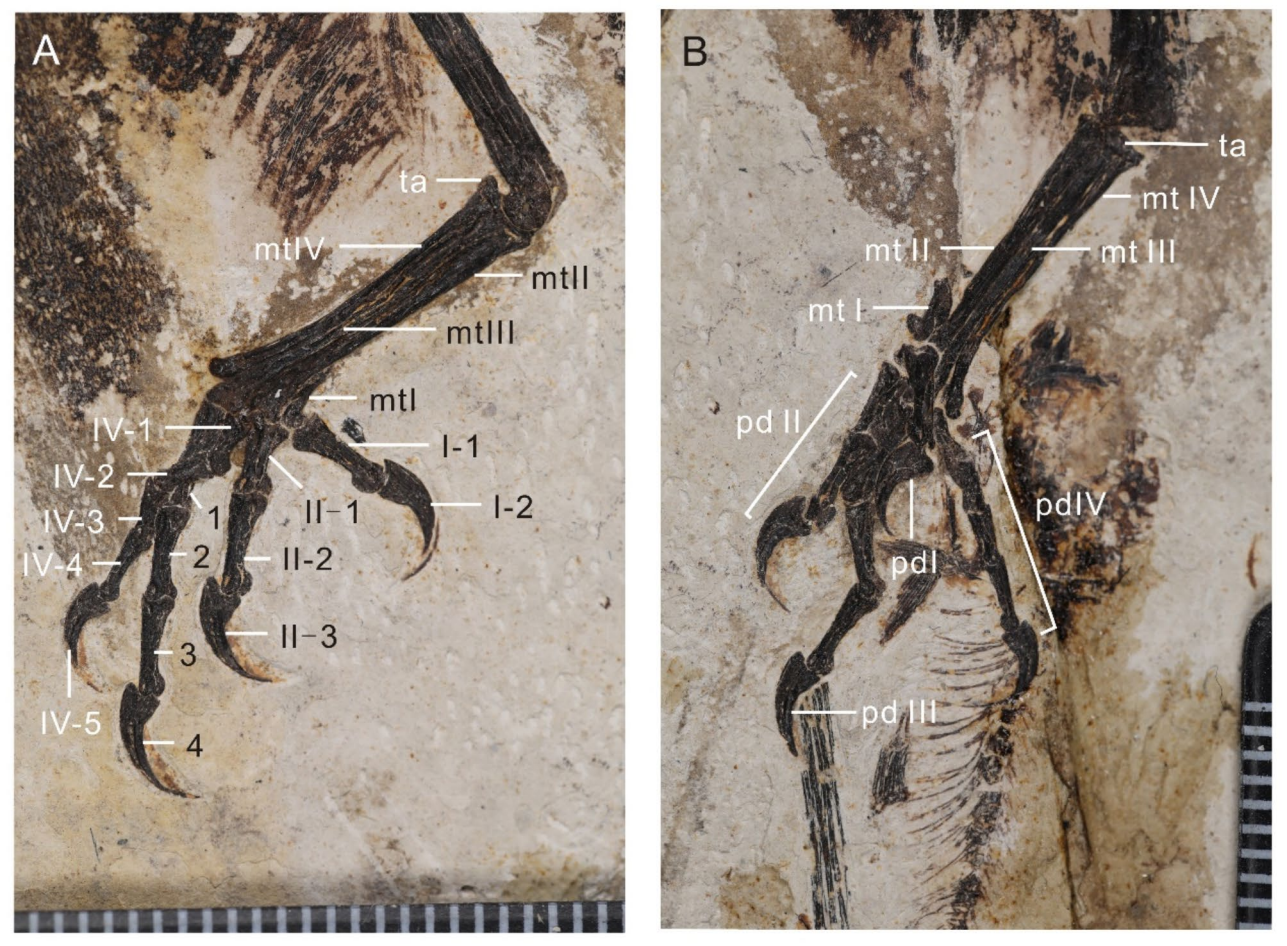
The feet of the holotype of *Neobohaiornis lamadongensis* gen.et sp. nov. (MHGU-0288). (**A**) left feet; (**B**) right feet. *mt I-IV* metatarsal I-IV, *pd I-IV* pedal digit I-IV, *ta* fused proximal tarsals, *I-1-2* pedal digit I-1-2, *II-1-3* pedal digit II-1-3, *III*-*1–4* pedal digit III-1-4, *IV-1-5* pedal digit IV-1-5.

The femora are very gently bowed caudally along their distal halves (Fig. 6). Proximodistal length of the femur measures 81% that of the tibiotarsus, which measures less than other bohaiornithids, such as *Parabohaiornis* 90%^17^, *Longusunguis* 86%^17^, *Bohaiornis* 85%^20^ and *Sulcavis* 87%^21^. The tibia shaft is straight; the proximal articular surface appears angled, similar to the condition in *Soroavisaurus*^37^, although this may be an artifact of preservation. A small and short fibular crest is present. The calcaneum and astragalus appear to be fused to the tibia. The proximal end of the fibula is expanded and bears a distinct tubercle for the m. *iliofibularis*. The fibula tapers distally; typically in enantiornithines, the fibula becomes very narrow and splint like just distal to the m. *iliofibularis* tubercle – this portion of the fibula appears to not be preserved on either side but was presumably present and did not reach the tarsals, based on comparison with other bohaiornithids (e.g., *Bohaiornis* LPM B00167^20^). The tarsals are fused to the metatarsals (Fig. 7). The proximodistal length of metatarsal III exceeds that of II and IV; metatarsal IV extends slightly farther distally than metatarsal II. The trochlea of metatarsal III is the widest and appears to be slightly angled such that the lateral condyle is distal to the medial condyle. The trochlea of metatarsal IV is the narrowest and appears to be reduced to a singular condyle, as in other enantiornithines. Metatarsal IV is mediolaterally narrower than metatarsals II and III. The entire tarsometatarsus tapers distally until the level of the proximal margin of metatarsal I, after which it increases in width, giving a waisted appearance. As in many other enantiornithines^6^, metatarsal I appears J-shaped with the articular surface for metatarsal II parallel to that for the hallux. Metatarsal I is preserved articulating with metatarsal II along the medial margin but this could be due to slight disarticulation. We interpret that the hallux was fully reversed, as in other known enantiornithines.

The pedal phalangeal formula is 2-3-4-5 (Fig. 7). Like other bohaiornithids, the pedal digits are robust with proportionately large, recurved claws. All the phalanges bear collateral ligament fossa and distally have symmetrical ginglymous articulations. Except for phalanx I and II of pedal digit III, all phalanges elongate distally within each digit, a feature associated with arboreality^35^. All the pedal digit ungulae bear neurovascular sulci in the lateral surface. The keratinous sheaths are preserved in all claws, increasing the length of the ungual by ~ 40%, similar to other bohaiornithids (e.g., *Parabohaiornis*^17^, *Bohaiornis*^20^, *Sulcavis*^21^) but less than in *Longusunguis*^28^.

### Feathers

**Fig. 8:**
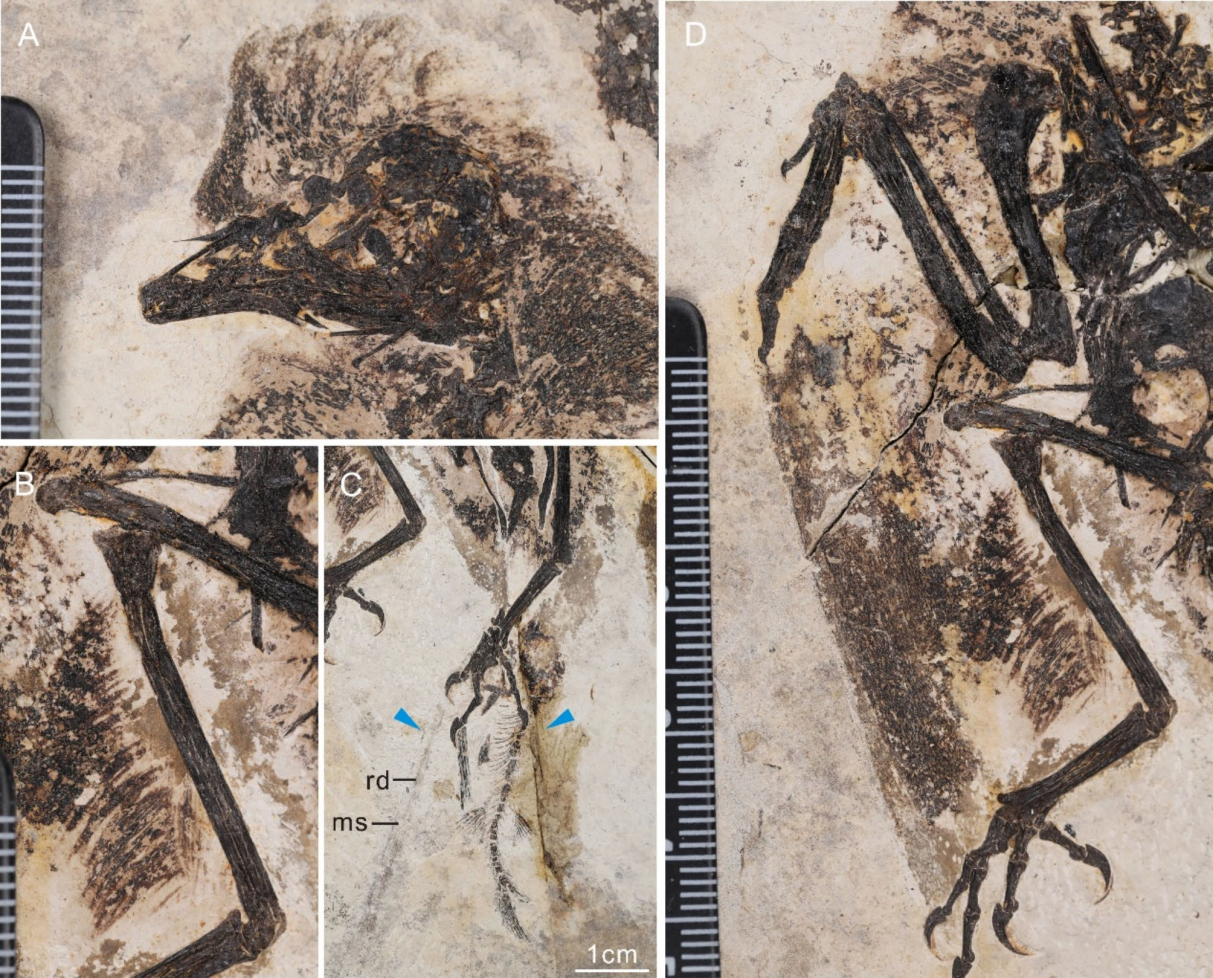
Plumage preserved in the holotype of *Neobohaiornis lamadongensis* gen.et sp. nov. (MHGU-0288). (**A**) apices of the feathers extending from the caudal margin of the external nares down the neck; (**B**) distribution of crural feathers along the left tibiotarsus; (**C**) A pair of rachis-dominated rectrices (blue arrows); (**D**) The primary wing feathers preserved on the left wing. *ms* medial stripe, *rd* rachis in ‘rachis-dominated’ feathers.

MHGU-0288 preserves a halo of feather traces extending from the caudal margin of the external nares down the neck, from the shoulder and pelvic regions, crural feathers, remiges, and a pair of rachis-dominated tail feathers (Fig. 8). The feathers on the head form a crest that is most likely the result of compression during diagenesis^38^. Similarly, the crural feathers also are oriented 90˚ from the tibiotarsus, but this was clearly not the natural orientation, providing further support that the crest-like morphology of cranial feathers is an artifact of preservation. However, the distribution of these feathers provides important information regarding the distribution of feathers on the skull, suggesting that the rostrum was covered in skin or scales. The primary wing feathers are approximately twice as long as the humerus, similar to the condition in CUGB P1202^25^. They appear to have rounded distal margins. The crural feathers are approximately half the length of the tibiotarsus. The distal ends of the rachis-dominated tail feathers are not preserved, as in *Bohaiornis* LPM B00167^20^ and bohaiornithid indet. CUGB P1202^25^.

### Phylogenetic analysis

**Fig. 9:**
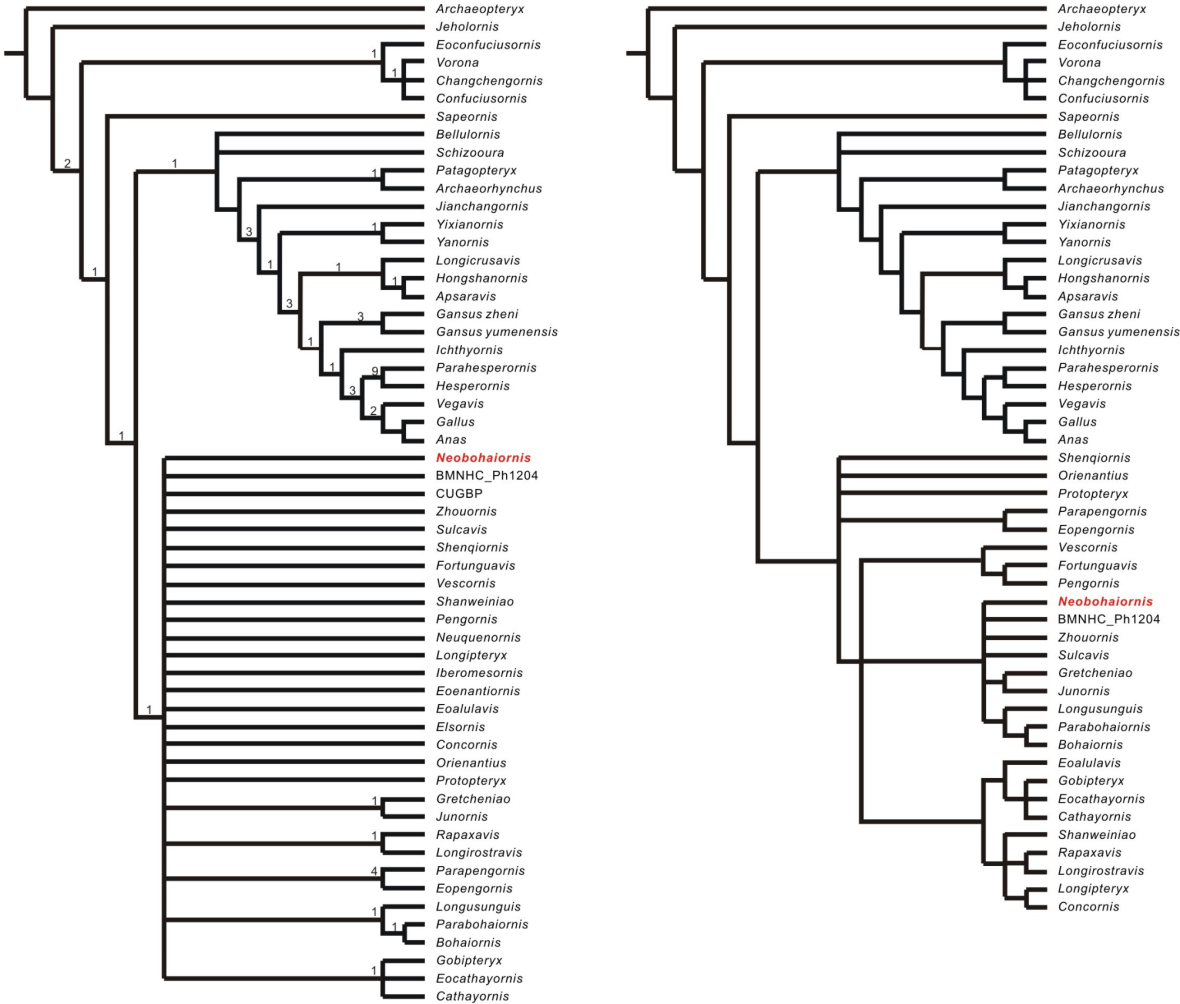
The relationships of *Neobohaiornis lamadongensis* gen.et sp. nov. (MHGU-0288). (**A**) The strict consensus tree from current phylogenetic analysis; (**B**) The reduced strict consensus tree after deactivate six unstable taxa from the same phylogenetic analysis. Bremer values are indicated.

The dataset consisted of 56 taxa and scored across 212 morphological characters (SI-SIII). The phylogenetic analysis produced 152 most parsimonious trees (MPTs), with 908 steps and consistency index = 0.322, retention index = 0.645, respectively. The analysis placing *Neobohaiornis* with other enantiornithines and forming a large polytomy (Fig. 9A). In order to improve resolution, a reduced consensus analysis performed using the “pruned trees” command embedded in TNT^39^. The five unstable taxa (*Elsornis*, *Eoenantiornis*, *Iberomesornis*, *Neuquenornis*, and bohaiornithid CUGBP-P1202) were pruned from the reduced consensus analysis (Fig. 9B). In the reduced consensus tree, *Neobohaiornis* forms a small polytomy with BMNHC-Ph1204, *Zhouornis*, *Sulcavis* and *Gretcheniao* + *Junornis* clade, which together constitute the outgroups of bohaiornithids, as in^13,24,28^. Within enantiornithines, *Shenqiornis*, *Orienantius*, *Protopteryx* and pengornithid clade (*Parapengornis* + *Eopengornis*) are resolved as the basal-most clade, as in some previous analyses^28,40,41^.

## Discussion

MHGU-0288 preserves morphologies that indicate it is a new taxon and a member of the diverse Jehol enantiornithine clade, the Bohaiornithidae. Compared to other previously known bohaiornithids, it preserves a few morphologies that are more similar to those present in crown birds (increased number of sacral vertebrae, reduced alular digit) that reveal the unique evolutionary history of this clade, as this lineage evolved more advanced flight capabilities in parallel to other enantiornithine clades and other early-diverging lineages of birds. In particular, MHGU-0288 reveals a highly reduced alular digit and an expanded first phalanx of the major digit, relatively to other bohaiornithids. The former also evolved in parallel to the Longipterygidae, in which *Rapaxavis* has the most reduced hand of any Early Cretaceous enantiornithine, having lost both its alular and major digit unguals in parallel to crownward ornithurines^14,15^. Although MHGU-0288 retains both its manual unguals, they are proportionately small compared to most enantiornithines, the alular digit is proportionately short, and the penultimate phalanx of the major digit is proportionately short. More tentatively, the poorly preserved synsacrum also appears to consist of more than seven sacral vertebrae, the typical number present in enantiornithines. This combination of features may suggest that this new taxon is relatively derived among bohaiornithids, but enantiornithine relationships are too poorly resolved for that level of detail to be discerned. A reduced manus and increased number of sacral vertebrae have evolved multiple times during the Cretaceous evolution of birds in parallel to crown birds^14,16^.

All major compound bones are fused in MHGU-0288 indicating it was mature or nearly so^40^. This indicates that its small body size compared to other bohaiornithids is a genuine feature. MHGU-0288 is approximately half the size of *Bohaiornis* IVPP V17963 (using humeral length as a proxy for size, which measures 51.95 mm in IVPP V17963 compared to 25 mm in MHGU-0288) (Table 2). Using the equations for estimating body mass published by Liu et al.^42^. based on humeral length, *Neobohaiornis* MHGU-0288 is estimated to have a body mass of 47 g compared to 162.46 g in *Bohaiornis* IVPP V17963. In contrast to *Neobohaiornis*, all other bohaiornithids are estimated to have body masses that exceed 100 g (Table 2). The discovery of *Neobohaiornis* therefore considerably increases the body size range and diversity of bohaiornithids. This hints at greater ecological diversity, although there currently exists no direct evidence concerning diet or ecological niche in this clade.

**Table 2:** Comparison of selected measurements (in mm) of bohaiornithid taxa.

Element/taxon	(1)*Bohaiornis*	(2)*Bohaiornis*	(3)*Parabohaiornis*	(4)*Longusunguis*	(5)*Sulcavis*	(6)*Shenqiornis*	(7)*Zhouornis*	(8)*Gretcheniao*	(9)*Neobohaiornis*
Skull	38.5*	47.1†	42.5	33.9*	42.4	41.6†	30.3	39.4†	26.7
Coracoid (L/W)	23.0/12.8	22.2/10.8†	21.9/12.3	24.2/12.2	24.8/12.1	26.2/10.7†	28.4/11.6†	29.3/9.5	6.4*/14.3*
Scapula	36.0	36.7	33.3	34.7	34.9	39.3	40.7	36.8†	16.3*
Humerus	47.0	52.0	43.4	40.3	46.5	46.6	50.6	49.7	25.4
Radius	45.4	48.5	40.3	40.5	47.7	45.8	50.1	49.2	21.5
Ulna	48.0	52.5	43.8	43.6	51.1	44.8	53.5	52.9	25.7
Alular mc	8.0	3.9	3.9	3.6	4.4	4.5	4.1	5.4	2.3
Major mc	21.3	19.5	16.5	16.8	20.7	21.7	19.5	25.9	11.6
Minor mc	22.7	21.0†	17.6	18.0	23.0	20.7	20.5	26.8	13.1
Alular ph I-1	9.5	19.0	8.1	7.1	9.6	10.0	9.1	11.2†	4.2
Major ph I-1	10.8	11.0	10.2	10.5	11.1	11.1	10.8	12.3	6.4
Major ph II-2	7.5	7.3	7.4	6.9	8.1	8.7	8.1	7.4	3.8
Minor ph III-1	5.5	6.7	5.5	5.2	6.7	6.9	5.6	6.6	3.1
Femur	39.0	42.6	36.0	35.8	41.3	38.8	44.5	41.1	22.2
Tibiotarsus	46.0	51.3	40.0	41.8	47.3	33*	52.1	49.6	27.4
Metatarsal I	5.7	5.3	4.3	5.1	5.0	7	5.2	4.8	2.8
Metatarsal II	20.8	–	17.1	19.6	21.6	22.4†	24.1	24.8	13.2
Metatarsal III	22.5	22.7	19.5	21.4	24.3	25	26.2	27.4	14.4
Metatarsal IV	21.8	–	18.1	20.2	22.6	22.8	24.8	25.2	14.0
Synsacrum	–	22.5*	14.3*	-	19.3	–	19.0†	15.7*	13.8
Pygostyle	18.5	19.2	18.0	22.8	19.6	–	17.3	20.6†	9.3
Body mass	136.58	162.46	118.96	104.62	134.57	117.06	155.21	150.46	47.00

(**1**) holotype of *Bohaiornis guoi*, LPM B00167^20^; (**2**) *Bohaiornis guoi*, IVPP V 17963^1^; (**3**)holotype of *Parabohaiornis martini*, VIPP V18691^17^; (**4**) holotype of *Longusunguis kurochkini*, IVPP V17694^17^; (**5**) holotype of *Sulcavis geeorum*, BMNH Ph 805^22^; (**6**) holotype of *Shenqiornis mengi*, DNHM D2950, mostly left side^22^; (**7**) holotype of *Zhouornis hani*, CNUVB-0903, right side^23,24^; (**8**) holotype of *Gretcheniao sinensis*, BMNH Ph 829^24^; (**9**) holotype of *Neobohaiornis lamadongensis*, MHGU-0288, left side. *L* length, *W* width, *mc* metacarpal, *Pp* phalanx. * indicates preserved length, † estimated. Body mass is estimated from humeral length using the equations by Liu et al.^40^.

MHGU-0288 also preserves new information pertaining to the plumage in bohaiornithids, which in most known specimens is poorly preserved with the exception of CUGB P1202^25^. These two specimens indicate the primary feathers of bohaiornithids were approximately twice the length of the humerus, with rounded feather margins, suggesting broad wings as in most other Early Cretaceous enantiornithines^43,44^. The feathers around the head in CUGB P1202 are poorly preserved with many feathers not preserved in situ^25^. In MHGU-0288 these feathers are well preserved forming a crest-like structure. In contrast to some previous interpretations^45^, we suggest this crest-like arrangement of feathers was formed post-mortem through taphonomic compression. This is strongly supported by the preserved orientation of the crural feathers, which like the crest are oriented 90˚ from the bone surface^39^. In the hindlimb, this is considered an unnatural position, given that the feathers are short, without asymmetry, and otherwise resembling contour feathers, and not flight feathers forming an aerodynamic surface. The crural feathers, as in CUGB P1202, are approximately half the length of the tibiotarsus^25^, which is shorter than in some enantiornithines^46^. MHGU-0288 also preserves traces of elongate, paired, rachis-dominated tail feathers, preserved also in the holotype of *Bohaiornis guoi*^20^ and CUGB P1202^25^. Unfortunately, these feathers are poorly preserved in all specimens so that the distribution of barbs and the shape of the racket cannot be determined. However, it strongly suggests that this tail morphology was consistent across the clade. In contrast, all evidence concerning the tail plumage in pengornithids, another diverse Jehol enantiornithine lineage, indicates that these birds had a pair of fully vaned rachis dominated tail feathers (streamers) together with a small, short, rectricial fan^29,47,48^. Among longipterygids, tail feathers are only weakly preserved in *Shanweiniao*^15,43,49^, which may suggest they had two pairs of rachis dominated tail feathers, similar to *Paraprotopteryx*^50^. This suggests that these major lineages differentiated themselves with distinct tail plumages, as well as through skeletal anatomy. Additional differences most likely also existed, such as disparate vocalizations, although these are impossible to determine in the fossil record, which consists primarily of hard tissues, and more rarely, soft tissues such as feathers.

The new specimen represents a new taxon that contributes to the considerable recognized diversity of bohaiornithid enantiornithines in the Jiufotang Formation, especially with regards to body size. This specimen highlights the skeletal adaptations, such as the reduction of the alular and major digit and possible increase in sacral vertebrae, evolved within this lineage, as they evolved more refined flight capabilities in parallel to other enantiornithine lineages, like the Longipterygidae. The new specimen preserves the best-known plumage of any bohaiornithid, which demonstrates that rachis-dominated tail feathers were likely widespread in this clade, as were crural feathers, and that the rostrum lacked feathers.

## Materials and methods

The new specimen (MHGU-0288) was discovered in the Lower Cretaecous Jiufotang Formation near Lamadong town outside Jianchang City in western Liaoning Province, China, and collected by The Museum of Hebei GEO University (MHGU), where the specimen is permanently reposited.

The phylogenetic matrix of Chiappe et al.^24^ was used to explore the phylogenetic position of *Neobohaiornis lamadongensis* through cladistics analysis. We assembled the dataset in Mesquite V3.1^51^, and conducted analyses in TNT version 1.1 software^52^. The analysis was run implementing a traditional (heuristic) search using the tree-bisection reconnection (TRB) swapping algorithm, retaining ten shortest trees out of 1000.

Institutional abbreviations: BMNHC, Beijing Museum of Natural History, Beijing, China; CUGB, China University of Geosciences, Beijing, China; IVPP, Institute of Vertebrate Paleontology and Paleoanthropology, Chinese Academy of Sciences, Beijing, China; LPM, Liaoning Paleontology Museum, Shenyang, Liaoning, China. MHGU, The Museum of Hebei GEO University, Shijiazhuang City, Hebei Province, China.

## Electronic supplementary material

Below is the link to the electronic supplementary material.

Supplementary Material 1

Supplementary Material 2

Supplementary Material 3

## Data Availability

Data is available in the manuscript or supplementary information file (SI-SIII).
