# Supplementary material for: A new diminutive species of bohaiornithid enantiornithine (Aves: Ornithothoraces) from the Lower Cretaceous Jehol Group, northern China
Source: Sci Rep. 2024 Dec 28;14:31363. doi: 10.1038/s41598-024-82869-8 (PMC11682239; doi:10.1038/s41598-024-82869-8)
Supplement: Supplementary file 1 — Supplementary Material 1 [file 41598_2024_82869_MOESM1_ESM.docx]

Supplementary 1: Character list used in the cladistics analyses

1. Premaxillae in adults: unfused (0); fused only rostrally (1); completely fused (2). (ORDERED)

2. Maxillary process of premaxilla: restricted to rostral portion (0); subequal or longer than facial contribution of maxilla (1).

3. Frontal process of premaxilla: short (0); relatively long, approaching rostral border of antorbital fenestra (1); very long, approaching the lacrimals (2). (ORDERED)

4. Premaxillary teeth: present throughout (0); present but rostral tip edentulous (1); present but restricted to rostral portion (2); absent (3).

5. Caudal margin of naris: far anterior than rostral border of antorbital fossa (0); nearly reaching or overlapping rostral border of antorbital fossa (1).

6. Naris longitudinal axis: considerably shorter than long axis of antorbital fossa (0); subequal or longer (1).

7. Maxillary teeth: present (0); absent (1).

8. Dorsal (ascending) ramus of maxilla: present with two fenestra (the promaxilllary and maxillary fenestra) (0); present with one fenestra (1); unfenestrated (2); ramus absent (3). (ORDERED)

9. Caudal margin of choana: located rostrally, not overlapping orbital region (0); displaced caudally, at same level or overlapping rostral margin of orbit (1).

10. Contact between palatine and maxilla/premaxilla: palatine contact maxilla only (0); contacts premaxilla and maxilla (1).

11. Jugal process of palatine: present (0); absent (1).

12. Ectopterygoid: present (0); absent (1).

13. Postorbital: present (0); absent (1).

14. Contact between postorbital and jugal: present (0); absent (1).

15. Quadratojugal: sutured to quadrate (0); joined through ligamentary articulation (1).

16. Lateral, round cotyla on mandibular process of quadrate (quadratojugal articulation): absent (0); present (1).

17. Squamosal incorporated into the braincase, forming a zygomatic process: absent (0); present (1).

18. Squamosal, zygomatic process: variably elongate, dorsally enclosing otic process of the quadrate and extending cranioventrally along shaft of this bone, dorsal head of quadrate not visible in lateral view (0); short, head of quadrate exposed in lateral view (1).

19. Frontal/parietal suture in adults: open (0); close, bones fully fused to one another (1).

20. Quadrate orbital process (pterygoid ramus): broad (0); sharp and pointed (1).

21. Quadrate pneumaticity: absent (0); present (1).

22. Quadrate: articulating only with squamosal (0); articulating with both prootic and squamosal (1).

23. Otic articulation of the quadrate: articulates with a single facet (squamosal) (0); articulates with two distinct facets (prootic and squamostal) (1); articulates with two distinct facets and quadrate differentiated into two heads (2). (ORDERED)

24. Quadrate distal end: with two transversely aligned condyles (0); with a triangular, condylar pattern, usually composed of three distinct condyles (1).

25. Eustachian tubes: paired, lateral, and well-separated from each other (0); paired, close to each other and to cranial midline or forming a single cranial opening (1).

26. Dentary teeth: present (0); absent (1).

27. Robustness of teeth relative to dentary: anteroposterior width of largest tooth crowns (measured at thickest portion of crown’s base) far less than half of dentary dorsoventral depth (0); close to half (or more) of dentary depth, i.e., 45%, or more (1). Taxa near the cutoff point are coded 0/1.

28. Dentary tooth implantation: teeth in individual sockets (0); teeth in a communal groove (1).

29. Symphysial portion of dentaries: unfused (0); fused (1).

30. Deeply notched rostral end of mandibular symphysis: absent (0); present (1).

31. Small ossification present at rostral tip of mandibular symphysis (intersymphysial ossification or “predentary bone”): absent (0); present (1).

32. Caudal margin of dentary: unforked, or with weakly developed dorsal ramus (0); strongly forked with dorsal and ventral rami approximately equal in caudal extent (1).

33. Meckel’s groove of mandible (medial side of mandible): not completely covered by splenial (0); covered by splenial, not exposed medially (1).

34. Rostral mandibular fenestra: absent (0); present (1).

35. Caudal mandibular fenestra: present (0); absent (1).

36. Articular pneumaticity: absent (0); present (1).

37. Atlantal hemiarches in adults: unfused (0); fused, forming a single arch (1).

38. One or more pneumatic foramina piercing centra of mid-cranial cervicals, caudal to level of parapophysis-diapophysis: present (0); absent (1).

39. Cervical vertebrae: variably dorsoventrally compressed, amphicoelous (“biconcave”: flat to concave articular surfaces) (0); cranial articular surface heterocoelous (i.e., mediolaterally concave, dorsoventrally convex), caudal articular surface flat or slightly concave (1); heterocoelous cranial (i.e., mediolaterally concave, dorsoventrally convex) and caudal (i.e., mediolaterally convex, dorsoventrally concave) articular surfaces (2). (ORDERED)

40. Prominent carotid processes in intermediate cervicals: absent (0); present (1).

41. Postaxial cervical epipophyses: prominent, projecting further back from postzygapophyses (0); weak, not projecting further back from postzygapophyses, or absent (1).

42. Keel-like ventral surface of cervical centra: absent (0); present (1).

43. Prominent (50% or more the height of centrum’s cranial articular surface) ventral processes of cervicothoracic vertebrae: absent (0); present (1).

44. Thoracic vertebral count: 13–14 (0); 11–12 (1); fewer than 11 (2). The transition between cervical and thoracic vertebrae is often difficult to identify, which makes counting these vertebrae problematic; we identify the first vertebra in articulation with a long costal rib as the first thoracic vertebra (ORDERED)

45. Thoracic vertebrae: at least part of series with subround, central articular surfaces (e.g., amphicoelous/opisthocoelous) that lack the dorsoventral compression seen in heterocoelous vertebrae (0); series completely heterocoelous (1).

46. Thoracic vertebrae, lateral side of centra: weakly or not excavated (0); deeply excavated by a groove (1); excavated by a broad fossa (2).

47. Cranial thoracic vertebrae, parapophyses: located in cranial part (0) or central part (1) of centra.

48. Sacral vertebrae, number ankylosed centra (synsacrum): less than 7 (0); 7 (1); 8 (2); 9 (3); 10 (4); more than 10 (5). (ORDERED)

49. Synsacrum, procoelous articulation with last thoracic centrum (deeply concave facet of synsacrum receives convex articulation of last thoracic centrum): absent (0); present (1).

50. Cranial vertebral articulation of first sacral vertebra: approximately equal in height and width (0); wider than high (1).

51. Degree of fusion of distal caudal vertebrae: fusion absent (0); few vertebrae partially ankylosed (intervening elements are well-discernable) (1); vertebrae completely fused into a pygostyle (2). (ORDERED)

52. Distal caudal vertebra prezygapophyses: elongate, exceeding the length of centrum by more than 25% (0); shorter (1); absent (2). (ORDERED)

53. Pygostyle: longer than or equal to the combined length of free caudals (0); shorter (1).

54. Cranial end of pygostyle dorsally forked: absent (0); present (1).

55. Cranial end of pygostyle with a pair of laminar, ventrally projected processes: absent (0); present (1).

56. Distal constriction of pygostyle: absent (0); present (1).

57. Ossified uncinate processes in adults: absent (0); present and free (1); present and fused (2).

58. Gastralia: present (0); absent (1).

59. Coracoid shape: rectangular to trapezoidal in profile (0); strutlike (1).

60. Coracoid-scapula articulation: “ball and socket” articulation (i.e., pit-shaped scapular cotyla developed on coracoid, and coracoidal tubercle developed on scapula) (0); scapular articular surface of coracoid convex (1); flat (2).

61. Scapula: articulated at omal end of coracoid (0); well below it (1).

62. Coracoid, humeral articular facet (glenoid): dorsal to acrocoracoid process (“biceps tubercle”) (0); ventral to acrocoracoid process (1).

63. Humeral articular facets of coracoid and scapula: placed in same plane (0); forming a sharp angle (1).

64. Coracoid, acrocoracoid: straight (0); hooked medially (1).

65. Coracoid, laterally compressed omal end with nearly aligned acrocoracoid process, humeral articular surface, and scapular facet, in dorsal view: absent (0); present (1).

66. Coracoid, procoracoid process: absent (0); present (1).

67. Coracoid, broad, deep fossa on dorsal surface (dorsal coracoidal fossa): absent (0); present (1).

68. Coracoid, supracoracoidal nerve foramen: centrally located (0); displaced toward (often as an incisure) medial margin (1); absent (i.e., nerve position is displaced so that it no longer passes through coracoid) (2). (ORDERED).

69. Coracoid, medial surface, strongly depressed elongate furrow (usually levelled with passage of n. supracoracoideus): absent (0); present (1).

70. Coracoid, width of the sternal end relative to the length along the shaft: approximately half or greater (0); between half to 1/3 (1); less than 1/3 (2).

71. Coracoid, sternal margin: convex (0); nearly straight (1); concave (2)

72. Coracoid, supracoracoid nerve foramen, location relative to dorsal coracoidal fossa: above fossa (0); inside fossa (1).

73. Coracoid, sternolateral corner: unexpanded (0); expanded (1); well developed squared-off lateral process (sternocoracoidal process) (2); present and with distinct omal projection (hooked) (3).

74. (244) Scapula and coracoid: fused (0); unfused (1).

75. Scapula, blade: straight (0); sagittally curved (1).

76. Scapula, length: shorter than humerus (0); as long as or longer than humerus (1).

77. (245) Scapula, acromion process length relative to length of humeral articular facet: less than half (0); nearly equivalent (1); longer but less than two times (2); more than two times longer (3). (ORDERED)

78. Scapula, acromion process: in lateral or costal view, strongly projecting craniodorsally, forming a large angle with proximal shaft (0); nearly parallel to shaft (1).

79. Scapula, costal surface of blade with prominent longitudinal furrow: absent (0); present (1).

80. Scapula, caudal end: blunt (may or may not be expanded) (0); sharply tapered (1).

81. Furcula: boomerang-shaped (0); V to Y-shaped (1); U-shaped (2).

82. Furcula, interclavicular angle: approximately 90° (0); less than 70° (1). The interclavicular angle is measured as the angle formed between three points: the omal ends of the rami and the center of the clavicular symphysis.

83. Furcula, dorsal and ventral margins: subequal in width (0); ventral margin distinctly wider than dorsal margin so that furcular ramus appears concave laterally (1).

84. Furcula, hypocleideum: absent (0); present as a tubercle or short process (1); present as elongate process approximately 30–50% the length of rami (2); hypertrophied, exceeding 50% the length of rami (3). (ORDERED)

85. Sternum: unossified (0); partially ossified, coracoidal facets cartilaginous (1); fully ossified (2).

86. Sternum, ossification: two flat bony plates (0); single, more or less flat element (1); single element, with slightly raised midline ridge (2); single element, with strongly projected carina (3).

87. Sternum, carina: near to, or projecting rostrally from, cranial border (0); not reaching cranial border (1).

88. Sternum, caudal margin, number of paired caudal trabeculae: none (0); one (1); two (2).

89. Sternum, outermost trabeculae: tips terminate cranial to caudal end of sternum (0); tips terminate at or approaching caudal end of sternum (1); tips extend caudally past the end of sternal midline (2).

90. Sternum, distal expansion of outermost trabecula: absent (0); present, simple bulb-like (1); fan-shaped (2); triangular–shaped with acute medial angle (3); branched (4).

91. Sternum, rostral margin broad and rounded: absent (0); present (1).

92. Sternum, coracoidal sulci spacing on cranial edge: widely separated mediolaterally (0); adjacent (1); crossed on midline (2). In taxa such as Eoalulavis in which the preserved sternum does not bear actual sulci, the placement of the coracoids (seemingly in their original place) can be used to infer their position relative to the sternum.

93. Sternum, costal facets: absent (0); present (1).

94. Sternum, caudal half, paired enclosed fenestra: absent (0); present (1).

95. Sternum, dorsal surface, pneumatic foramen (or foramina): absent (0); present (1).

96. (243) Sternum, outermost trabecula: mainly parallel to long axis of sternum (0); clearly directed caudo-laterally (1).

97. Humerus, proximal and distal ends: twisted (0); nearly co-planar (1)

98. Humerus, head: concave cranially and convex caudally (0); globe shaped, craniocaudally convex (1).

99. Humerus, proximal margin of head is concave in its central portion, rising ventrally and dorsally: absent (0); present (1).

100. Humerus, proximocranial surface, well-developed circular fossa on midline: absent (0); present (1).

101. Humerus, transverse ligamental groove: absent (0); present (1).

102. Humerus, ventral tubercle projected caudally, separated from humeral head by deep capital incision: absent (0); present (1).

103. Humerus, pneumatic fossa in caudoventral corner of proximal end: absent or rudimentary (0); well developed (1).

104. Humerus, deltopectoral crest: projected dorsally (the plane of the crest is coplanar to cranial surface of humerus) (0); projected cranially (1).

105. Humerus, deltopectoral crest width: less than shaft width (0); approximately same width (1); prominent and subquadrangular (i.e., subequal length and width) (2).

106. Humerus, deltopectoral crest, distal end recedes abruptly with the humeral shaft: present (0); absent (1)

107. Humerus, deltopectoral crest: imperforated (0); perforated by a fenestra (1).

108. Humerus, bicipital crest: little to no cranial projection (0); developed as cranial projection relative to shaft surface in ventral view (1); hypertrophied, rounded tumescence (2).

109. Humerus, distal end of bicipital crest, pit-shaped fossa for muscular attachment: absent (0); craniodistal on bicipital crest (1); directly ventrodistal at tip of bicipital crest (2); caudodistal, variably developed as a fossa (3).

110. Humerus, demarcation of muscle origins (e.g., m. extensor metacarpi radialis) on the dorsal edge of the distal humerus: no indication (0); a pit or a tubercle (1); a variably projected scar-bearing tubercle (dorsal supracondylar process) (2).

111. Humerus, well-developed brachial depression on cranial face of distal end: absent (0); present (1).

112. Humerus, well-developed olecranon fossa on caudal face of distal end: absent (0); present (1).

113. Humerus, groove for passage of m. scapulotriceps: absent (0); present (1).

114. Humerus, m. humerotricipitalis groove: absent (0); present as a well-developed ventral depression contiguous with the olecranon fossa (1).

115. Humerus, distal margin: approximately perpendicular to long axis of shaft (0); strongly angled ventrally (ventrodistal margin projected significantly distal to dorsodistal margin) (i.e., well-projected flexor process (1).

116. Humeral distal condyles: mainly located on distal aspect (0); on cranial aspect (1).

117. Humerus, long axis of dorsal condyle: at low angle to humeral axis, proximodistally oriented (0); at high angle to humeral axis, almost transversely oriented (1).

118. Humerus, distal condyles: subround, bulbous (0); weakly defined, “straplike” (1).

119. Humerus, ventral condyle: length of long axis less than the same measure in dorsal condyle (0); same or greater (1).

120. Ulna: shorter than humerus (0); nearly equivalent to or longer than humerus (1).

121. Ulna: mid-shaft relative width: radial-shaft/ulnar-shaft ratio larger than 0.70 (0); smaller than 0.70 (1).

122. Ulna, cotylae: dorsoventrally adjacent (0); widely separated by deep groove (1).

123. Ulna, dorsal cotyla strongly convex: absent (0); present (1).

124. Ulna, bicipital scar: absent (0); developed as slightly raised scar (1); developed as conspicuous tubercle (2).

125. Ulna, proximal end with well-defined area for insertion of m. brachialis anticus: absent (0); present (1).

126. Ulna, semilunate ridge on dorsal condyle: absent (0); present (1).

127. Radius, long longitudinal groove on ventrocaudal surface of shaft: absent (0); present (1).

128. Ulnare: heart-shaped with little differentiation into short rami (0); U-shaped to V-shaped, well-developed rami (1).

129. Semilunate carpal and proximal ends of metacarpals in adults: unfused (0); semilunate fused to the alular (I) metacarpal (1); semilunate fused to the major (II) and minor (III) metacarpals (2); fusion of semilunate and all metacarpals (3). Juvenile specimens are scored as “?” to account for the possibility of ontogenetic change.

130. Semilunate carpal, position relative to alular metacarpal (I): over entire proximal surface (0); over less than one-half proximal surface or no contact present (1).

131. Carpometacarpus, proximal ventral surface: flat (0); raised ventral projection contiguous with minor metacarpal (1); pisiform process forming a distinct peg-like projection (2).

132. Carpometacarpus, proximoventral surface, supratrochlear fossa deeply excavating proximal surface of pisiform process: absent (0); present (1).

133. Alular metacarpal (I), round-shaped: absent (0); present (1).

134. Alular metacarpal (I), extensor process: absent (0); tip barely (1) or conspicuously (2) surpasses cranial margin of distal articular facet. (ORDERED)

135. Alular metacarpal (I), distal articulation with proximal phalanx: ginglymoid (0); shelf (1); ball-like (2).

136. Minor metacarpal (III), craniocaudal diameter as percentage of same dimension of major metacarpal (II): approximately equal or greater than 50% (0); less than 50% (1).

137. Alular digit (I), proximal phalanx: longer than proximal phalanx of major digit (II) (0); shorter than or equivalent to proximal phalanx of major digit (II) (1).

138. Intermetacarpal process (or tubercle) on major metacarpal (II): absent (0); present (1).

139. Intermetacarpal space: absent or very narrow (0); at least as wide as maximum width of minor metacarpal (III) shaft (1).

140. Intermetacarpal space: reaches proximally as far as distal end of alular metacarpal (I) (0); terminates distal to end of alular metacarpal (I) (1).

141. Distal end of metacarpals: unfused (0); partially or completely fused (1).

142. Minor metacarpal (III) projecting distally more than major metacarpal (II): absent (0); present (1).

143. Alular digit (I), proximal phalanx, distal extension relative to major metacarpal (II): beyond distal end of major metacarpal (II) (0); approximately equal in distal extension (1); shorter than distal end but beyond half of major metacarpal (II) (2); terminating less than half of major metacarpal (II) (3). (ORDERED)

144. Proximal phalanx of major digit (II): round-shaped cross section (0); flat and craniocaudally expanded (1).

145. Intermediate phalanx of major digit (II): longer than proximal phalanx (0); shorter than or equivalent to proximal phalanx (1).

146. Ungual phalanx of major digit (II): present (0); absent (1).

147. Ungual phalanx of major digit (II): larger or subequal to other manual unguals (0); smaller than alular ungual but larger than ungula phalanx of minor (III) digit (ungual of minor digit may or may not be present) (1); smaller than unguals of alular and minor digits (2).

148. Ungual phalanx of minor digit (III): present (0); absent (1).

149. Manus, relative length: length of semilunate carpal + major metacarpal and digit longer than humerus (0); subequal (1); shorter (2). (ORDERED)

150. Intermembral index = (length of humerus + ulna)/(length of femur + tibiotarsus): less than 0.7 (0); between 0.7 and 0.9 (1); between 0.9 and 1.1 (2); greater than 1.1 (3).

151. Pelvis, bone fusion at level of acetabulum: unfused or partial fusion (0); completely fused (1). Juvenile specimens with unfused pelvis are scored “?” to allow for ontogenetic fusion.

152. Ilium/ischium, distal co-ossification to completely enclose ilioischiadic fenestra: absent (0); present (1).

153. Ilium, midline proximity of preacetabular wings: separated (may exist cartilaginous connection) (0); co-ossified, dorsal closure of “iliosynsacral canals” (1).

154. Preacetabular pectineal process (preacetabular tubercle of Baumel): absent (0); present (1).

155. Pelvis, acetabulum proportions: large acetabulum, acetabulum/ilium length ratio greater than 0.11 (0); small acetabulum, same proportion equal or smaller than 0.11 (1).

156. Prominent antitrochanter: caudally directed (0); caudodorsally directed (1).

157. Ilium, postacetabular process: deep (0), more than 50% of depth of preacetabular wing at level of acetabulum; shallow (1), less than 50%.

158. Ilium, brevis fossa: present (0); absent (1).

159. Ischium, relative length: two-thirds or less the length of pubis (0); more than two-thirds the length of pubis (1).

160. Ischium, obturator process: prominent (0); reduced or absent (1). 1

161. Ischium, proximodorsal (or proximocaudal) process: absent (0); present (1).

162. Pubis, orientation of proximal portion: cranially to subvertically oriented (0); retroverted, separated from main synsacral axis by a 45–65° angle (1); more or less parallel to ilium and ischium (2). (ORDERED)

163. Ilium, pubic pedicel very compressed laterally and hook-like: absent (0), present (1).

164. Pubis, shaft laterally compressed throughout its length: absent (0); present (1).

165. Pubis, pubic apron: present (0); absent (absence of symphysis) (1).

166. Pubis, distal foot: flaring into simple round shape (0); triangular shape with pointed caudal tip and caudoventally directed with respect to distal pubic shaft (1); caudal tip recurved caudodorsally with respect to distal pubic shaft (2); absent (3).

167. Femur, distinct fossa for capital ligament: absent (0); present (1).

168. Femur, neck: present (0); absent (1).

169. Femur, anterior trochanter: separated from greater trochanter (0); fused to it, forming a trochanteric crest with laterally curved edge (1); fused to it, forming a trochanteric crest with flattened edge (2).

170. Femur, trochanteric crest: projects proximally beyond femoral head (0); equal in proximal projection (1); does not project beyond femoral head (2).

171. Femur, posterior trochanter: present, developed as slightly projected tubercle or flange (0); hypertrophied, “shelf-like” conformation (1); absent (2).

172. Femur, prominent patellar groove: absent (0); present as continuous extension onto distal shaft (1); present and separated from shaft by slight ridge, giving it a pocketed appearance (2).

173. Femur, lateral distal end: ectocondylar tubercle and lateral condyle separated by deep notch (0); ectocondylar tubercle and lateral condyle contiguous but without developing a tibiofibular crest (1); tibiofibular crest present, defining laterally a fibular trochlea (2). (ORDERED)

174. Femur, popliteal fossa distally bounded by a complete transverse ridge: absent (0); present (1).

175. Tibia, calcaneum, and astragalus: unfused or poorly co-ossified (sutures still visible) (0); complete fusion of tibia, calcaneum, and astragalus (1).

176. Tibia, round proximal articular surface: absent (0); present (1).

177. Tibia, proximal articular surface: flat (0); angled so that medial margin is elevated with respect to lateral margin (1).

178. Tibiotarsus, proportions: tibiotarsus length/tarsometatarsus length equals 2 or more (0); between 2 and 1.6 (1); smaller than 1.6 (2). When distal tarsals are not fused with metatarsals, metatarsal III length is used.

179. Tibiotarsus, cnemial crests: absent (0); present, one (1); present, two (2).

180. Tibia, caudal extension of articular surface for tarsals/tarsometatarsus: absent, articular surface restricted to distalmost edge of caudal surface (0); well-developed caudal extension, sulcus cartilaginis tibialis ([Baumel & Witmer, 1993](https://scholar.google.com/scholar_lookup?title=Osteologia&author=Baumel&publication_year=1993)), distinct surface extending up caudal surface of tibiotarsus (1); with well-developed, caudally projecting medial and lateral crests (2). (ORDERED)

181. Tibiotarsus, extensor canal: absent (0); present as emarginate groove (1); groove bridged by ossified supratendinal bridge (2). (ORDERED)

182. Tibibiotarsus, condyles, cranial projection: medial condyle projecting farther cranially than lateral condyle (0); equal in cranial projection (1).

183. Tibiotarsus, condyles, relative mediolateral width: medial condyle wider (0); approximately equal (1); lateral condyle wider (2). (ORDERED).

184. Tibiotarsus, condyles, intercondylar groove: mediolaterally broad, approximately 1/3 with of anterior surface (0); less than 1/3 width of anterior surface (1).

185. Tibiotarsus, condyles: gradual sloping of condyles towards tibiotarsal midline of tibiotarsus (0); no tapering of either condyle (1).

186. Fibula, proximal end: prominently excavated by medial fossa (0); nearly flat (1).

187. Fibula, tubercle for m. iliofibularis: craniolaterally directed (0); laterally directed (1); caudolaterally or caudally directed (2). (ORDERED)

188. Fibula, distal end reaching proximal tarsals: present (0); absent (1).

189. Distal tarsals in adults: free (0); completely fused to the metatarsals (1). Juvenile specimens are scored as “?” in order to account for the possibility of ontogenetic change.

190. Metatarsals II–IV, intermetatarsal fusion: absent or minimal co-ossification (0); partial fusion, sutural contacts easily discernible (1); completely or nearly completely fused, sutural contacts absent or poorly demarcated (2). (ORDERED)

191. Metatarsal V: present (0); absent (1).

192. Metatarsal III, proximal end: co-planar with metatarsals II and IV (0); plantarly displaced with respect to metatarsals II and IV (1).

193. Tarsometatarsus, proximal vascular foramen and/or foramina between metatarsals III and IV: absent (0); one (1); two (2).

194. Tarsometatarsus, intercotylar eminence: absent (0); present, low and rounded (1); present, high and peaked (2).

195. Tarsometatarsus, projected surface and/or grooves on proximocaudal surface (associated with the passage of tendons of pes flexors; hypotarsus): absent (0); developed as caudal projection with flat caudal surface (1); at least one groove enclosed by bone caudally (2). (ORDERED)

196. Tarsometatarsus, plantar surface: flat (0); excavated (1).

197. Tarsometatarsus, distal vascular foramen completely enclosed by metatarsals III and IV: absent (0); present (1).

198. Metatarsal I: straight (0); J-shaped, with short projection (articulation of hallux) extending medially (1); J-shaped; articulation of hallux extending caudally (2); distal half of metatarsal I is laterally deflected so that laterodistal surface is concave (3).

199. Metatarsal II tubercle (associated with the insertion of the tendon of the m. tibialis cranialis): absent (0); present, approximately centered on proximodorsal surface of metatarsal II (1); present, developed on lateral surface of metatarsal II, at contact with metatarsal III or on lateral edge of metatarsal III (2).

200. Metatarsal II, distal plantar surface, fossa for metatarsal I: absent (0); shallow notch (1); conspicuous ovoid fossa (2). (ORDERED)

201. Relative position of metatarsal trochleae: trochlea III more distal than trochleae II and IV (0); trochlea III at same level as trochlea IV, both more distal than trochlea II (1); trochlea III at same level as trochleae II and IV (2); distal extent of trochlea III intermediate to trochlea IV and II where trochlea IV projects furthest distally (3).

202. Metatarsal II, distal extent of metatarsal II relative to metatarsal IV: approximately equal in distal extent (0); metatarsal II shorter than metatarsal IV but reaching distally farther than base of metatarsal IV trochlea (1); metatarsal II shorter than metatarsal IV, reaching distally only as far as base of metatarsal IV trochlea (2).

203. Tarsometatarsus, trochlea in distal view: aligned in a single plane (0); metatarsal II slightly displaced plantarly with respect to III and IV (1); metatarsal II strongly displaced plantarly in respect to III and IV, such that there is little or no overlap in medial view (2).

204. Metatarsal II, trochlea of metatarsal II broader than trochlea of metatarsal III: absent (0); present (1).

205. Metatarsal III, trochlea in plantar view, proximal extent of lateral and medial edges of trochlea: trochlear edges approximately equal in proximal extent (0); medial edge extends farther (1).

206. Distal end of metatarsal II strongly curved medially: absent (0); present (1).

207. Phalanx in digit IV; second and third phalanges reduced and significantly shorter than fourth phalanx: absent (0), present (1), present, but with proximal phalanx reduced to be nearly equal in length with second and third phalanx (2).

208. Digit IV phalanges in distal view, medial trochlear rim enlarged with respect to lateral trochlear rim: absent (0); present, lateral trochlea reduced to a rounded peg (1).

209. Pes, proximal phalanx of hallux is the longest non-ungual phalanx: absent (0); present (1).

210. Size of claw of hallux relative to other pedal claws: shorter, weaker, and smaller (0); similar in size (1); longer, more robust, and larger (2).

211. Alula: absent (0); present (1).

212. Fan-shaped feathered tail composed of more than two elongate retrices: absent (0); present (1).
